# Role of Individual Subunits of the *Neurospora crassa* CSN Complex in Regulation of Deneddylation and Stability of Cullin Proteins

**DOI:** 10.1371/journal.pgen.1001232

**Published:** 2010-12-02

**Authors:** Jiyong Wang, Qiwen Hu, Huijie Chen, Zhipeng Zhou, Weihua Li, Ying Wang, Shaojie Li, Qun He

**Affiliations:** 1 State Key Laboratory of Agrobiotechnology, College of Biological Sciences, China Agricultural University, Beijing, China; 2 Institute of Basic Medical Sciences, National Center of Biomedical Analysis, Beijing, China; 3 Key Laboratory of Systematic Mycology and Lichenology, Institute of Microbiology, Chinese Academy of Sciences, Beijing, China; Yale University, United States of America

## Abstract

The Cop9 signalosome (CSN) is an evolutionarily conserved multifunctional complex that controls ubiquitin-dependent protein degradation in eukaryotes. We found seven CSN subunits in *Neurospora crassa* in a previous study, but only one subunit, CSN-2, was functionally characterized. In this study, we created knockout mutants for the remaining individual CSN subunits in *N. crassa*. By phenotypic observation, we found that loss of CSN-1, CSN-2, CSN-4, CSN-5, CSN-6, or CSN-7 resulted in severe defects in growth, conidiation, and circadian rhythm; the defect severity was gene-dependent. Unexpectedly, CSN-3 knockout mutants displayed the same phenotype as wild-type *N. crassa*. Consistent with these phenotypic observations, deneddylation of cullin proteins in *csn-1*, *csn-2*, *csn-4*, *csn-5*, *csn-6*, or *csn-7* mutants was dramatically impaired, while deletion of *csn-*3 did not cause any alteration in the neddylation/deneddylation state of cullins. We further demonstrated that CSN-1, CSN-2, CSN-4, CSN-5, CSN-6, and CSN-7, but not CSN-3, were essential for maintaining the stability of Cul1 in SCF complexes and Cul3 and BTB proteins in Cul3-BTB E3s, while five of the CSN subunits, but not CSN-3 and CSN-5, were also required for maintaining the stability of SKP-1 in SCF complexes. All seven CSN subunits were necessary for maintaining the stability of Cul4-DDB1 complexes. In addition, CSN-3 was also required for maintaining the stability of the CSN-2 subunit and FWD-1 in the SCF^FWD-1^ complex. Together, these results not only provide functional insights into the different roles of individual subunits in the CSN complex, but also establish a functional framework for understanding the multiple functions of the CSN complex in biological processes.

## Author Summary

Protein degradation is precisely controlled in cells. The ubiquitin-mediated protein degradation pathway is highly conserved in eukaryotes, and the activity of ubiquitin ligases is regulated by the Cop9 signalosome (CSN), a multisubunit complex that is evolutionarily conserved from yeast to humans. Determining how the CSN complex functions biologically is crucial for understanding regulation of the ubiquitin-mediated protein degradation pathway. The filamentous fungus *N. crassa* is commonly used to study protein degradation. Its CSN complex contains seven subunits (CSN-1 to CSN-7). In this study, we generated knockout mutants of individual CSN subunits and observed the phenotypes of each mutant. We demonstrated that six of the seven CSN subunits were essential for cleaving the ubiquitin-like protein Nedd8 from cullin proteins (which act as scaffolds for ubiquitin ligases). In contrast, loss of the CSN-3 subunit had no effect on cullin neddylation. We also found that each CSN subunit had distinct roles in maintaining the stability of key components of cullin-based ubiquitin ligases. In summary, we systematically investigated the unequal contributions of CSN subunits to deneddylation and the maintenance of cullin-based ubiquitin ligases in *N. crassa*. Our work establishes a framework for understanding the function of CSN subunits in other eukaryotes.

## Introduction

The Cop9 signalosome (CSN) is a multiprotein complex that was initially discovered in *Arabidopsis thaliana* as an important regulator of photomorphogenesis, and was later found to participate in a wide range of processes in eukaryotes [1]. The CSN usually contains eight subunits (CSN1–CSN8) in higher eukaryotes, and each CSN subunit has evolutionarily conserved counterparts in the 26S proteasome lid complex and eukaryotic translation initiation factor 3 (eIF3) [2], [3]. All known CSNs regulate ubiquitin-dependent protein degradation [4].

The ubiquitin–proteasome system is the major pathway responsible for the degradation of intracellular proteins. In this pathway, proteins targeted for rapid degradation are conjugated to ubiquitin, a small conserved protein with 76 amino acids [5]. The attachment of ubiquitin to its target proteins is mediated by a cascade of enzymatic reactions involving the ubiquitin-activating enzyme (E1), ubiquitin-conjugating enzyme (E2), and ubiquitin ligase (E3). After recruiting the specific substrate, the ubiquitin ligase (E3) complex bridges the targeted protein and E2-ubiquitin to form a polyubiquitinated protein, which is subsequently degraded by the 26S proteasome [6]. Cullin-RING ubiquitin ligases (CRLs) are the major group of E3s. A typical CRL complex consists of a cullin subunit (Cul1, Cul3, or Cul4), a RING protein (Hrt1/Roc1/Rbx1), an adaptor protein (Skp1 in SCF complexes, DDB1 in Cul4-based E3), and a substrate-recognition subunit such as F-box proteins (FBPs) in SCF complexes [7], BTB proteins in Cul3-type E3 complexes [8], and DCAFs in Cul4-DDB1 E3 complexes [9]–[11]. In eukaryotic systems, CRLs play essential roles in many processes, including cell division, cell proliferation, cell differentiation, and circadian clock function [12]. The CRLs are activated by the neddylation process, in which Nedd8, a ubiquitin-like protein, is attached to a conserved lysine site on cullin proteins. The neddylated cullin may accelerate assembly of the CRL E3 complex, which promotes the ubiquitination of its substrate. The CSN negatively regulates the activity of CRLs by deneddylation, in which Nedd8 is cleaved from cullin proteins [4], [13]. Disruption of CSN subunits generally causes hyperneddylation of Cul1 and other cullins in many organisms [4], [12]–[16]. Genetic evidence indicates that CSN promotes CRL-mediated degradation of substrates *in vivo* [4], [17], [18]. Therefore, CSN has been proposed to mediate the assembly/disassembly of CRLs [17], and recent studies demonstrate that a major function of the CSN complex is to control the stability of CRL ubiquitin ligases *in vivo* [19]–.

Eight subunits of CSN are present in higher eukaryotes, as well as in *Dictyostelium discoideum* and *Aspergillus nidulans*. Interestingly, three CSN subunits are missing in *Saccharomyces cerevisiae* (CSN4, CSN6, and CSN8) [22] and two are missing in *Schizosaccharomyces pombe* (CSN6 and CSN8) [23], while both *Caenorhabditis elegans* [24] and *N. crassa* [20] lack CSN8. The absence of one or more CSN complex subunits in lower eukaryotes suggests that the composition of the CSN complex is species specific. The role of each CSN subunit may differ in some species, and different CSN subunits may not contribute equally to the function of the CSN complex. For example, in *S. pombe*, mutants with different CSN subunits deleted display distinct phenotypes [14]. In *Drosophila melanogaster*, both *csn4* and *csn5* mutants have defects in oogenesis and embryo patterning, as well as larval lethality. However, *csn4-*null flies exhibit molting defects, while *csn5-*null flies develop melanotic tumors [25]. In addition, *csn4* and *csn5* mutants show different gene expression patterns [26].

Compared to yeast and higher organisms, the functions and roles of the individual CSN subunits are poorly understood in filamentous fungi. To date, only four out of the eight subunits in *A. nidulans* and one out of the seven subunits in *N. crassa* have been investigated [20], [27]. Furthermore, the functions of individual CSN subunits within the CSN complex are largely unknown in eukaryotes. Therefore, to further understand the functions and roles of the CSN complex with regard to the regulation of CRLs, we performed a systematic functional analysis of each subunit in the *N. crassa* CSN complex.

## Results

### *N. crassa* CSN complex composed of seven subunits

Recently, the purification of Myc-His-CSN-2 protein expressed in a *csn-2^KO^* mutant led to the identification of the *N. crassa* CSN complex, which contains seven subunits (CSN-1 to CSN-7a) (Table S1) [20]. Like most other fungi, the *N. crassa* genome does not encode a *csn-8*–like gene. Protein sequence alignment indicated that the *N. crassa* CSN subunits were more closely related to the CSN subunits of animals and *A. thaliana* than to those of yeast. Bioinformatics analyses further showed that *N. crassa* CSN-3 was the least-conserved subunit in the CSN complex, with a lower *e*-value PCI (proteasome, COP9 signalosome, eukaryotic initiation factor 3) domain. Similarly, in *A. nidulans*, CsnC (CSN-3) and CsnH (CSN-8) are also less-conserved subunits in the CSN complex [28]. Interestingly, we also found that there is another *csn-7*–like gene (*csn-7b*, NCU02813) in the *N. crassa* genome that encodes a protein with a highly conserved PCI domain. However, this hypothetical protein was not detected in purification products of the CSN complex [20]. Thus, the *N. crassa* CSN complex consists of seven subunits: five PCI domain proteins (CSN-1, CSN-2, CSN-3, CSN-4, and CSN-7) and two MPN (Mpr-Pad1-N-terminal) domain proteins (CSN-5 and CSN-6) (Table S1).

### Deletion of individual CSN subunits led to different defects in growth and conidiation

Of the seven CSN subunits in *N. crassa*, only CSN-2 has been functionally characterized [20]. To systematically analyze the function of each CSN subunit, we generated deletion mutants for each of the remaining six *csn* genes by gene replacement with a hygromycin resistance gene (*hph*). As with the *csn-2^KO^* mutant, we obtained homokaryotic deletion strains of each single *csn* gene, indicating that none of the seven *csn* genes was essential for the cell viability of *N. crassa*. However, attempts to generate homokaryotic mutants for the *csn-7*–like gene *csn-7b* (NCU02813) were unsuccessful, suggesting that this gene is essential for cell viability. The failure to generate *csn-7b* homokaryotic deletion mutants suggests a functional difference between *csn-7b* and the other *csn* genes. This result, together with the absence of CSN-7b in Myc-His-CSN-2 purification products, further suggests that the product of the *csn-7b* gene is not a component of the *N. crassa* CSN complex.

**Figure 1:**
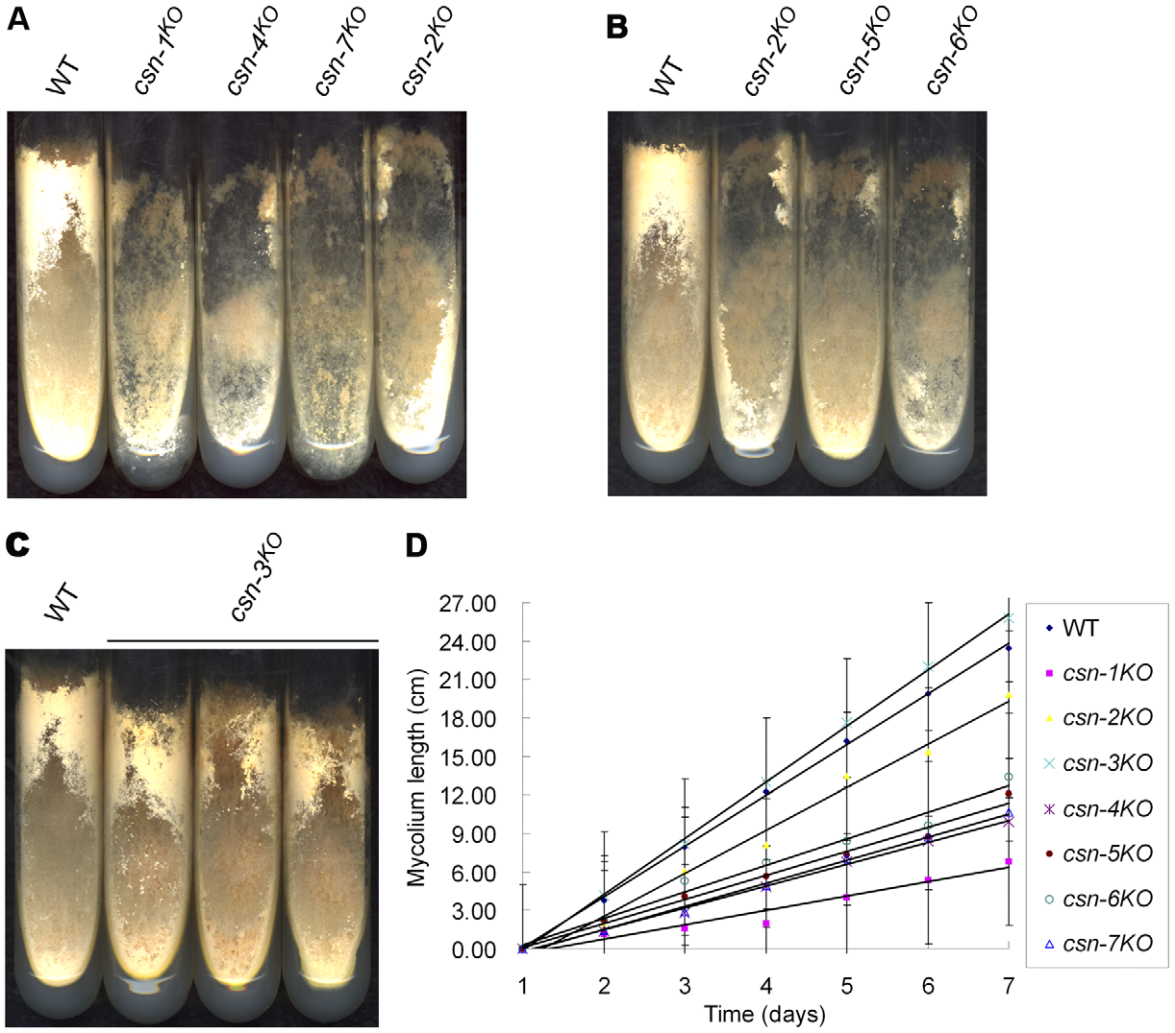
Effect of disrupting individual *csn* subunits on growth and developmental phenotypes. (A) Wild-type and *csn-1*, *csn-4*, and *csn-7* mutant strains growing on minimal slants. *csn-1*, *csn-4*, and *csn-7* mutant strains produced significantly less conidia and aerial hyphae than the wild-type strain and were weaker than the *csn-2* mutant. (B) *csn-2^KO^*, *csn-5^KO^*, and *csn-6^KO^* had similar phenotypes on minimal slants. (C) Disruption of *csn-3* had little effect on growth and development on minimal slants. (D) Growth rate of the wild-type strain and seven *csn* subunit mutants, measured at 25°C using the race tube assay in constant darkness after one day of light treatment.

We recently showed that the CSN-2 subunit plays important roles in *N. crassa* growth and development [20]. The *csn-1^KO^, csn-4^KO^*, and *csn-7^KO^* strains produced fewer conidia and aerial hyphae on slants than the *csn-2^KO^* mutant (Figure 1A), while the *csn-5^KO^* and *csn-6^KO^* strains exhibited similar phenotypes to the *csn-2* mutant (Figure 1B). These results suggest that these subunits are important for *N. crassa* development. In addition, the growth rates of the *csn-1^KO^*, *csn-2^KO^*, *csn-4^KO^*, *csn-5^KO^*, *csn-6^KO^*, and *csn-7^KO^* strains were markedly slower than that of the wild-type strain at normal temperature (Figure 1D). The severity of the growth defects was gene dependent. Compared with that of the *csn-2^KO^* mutant, the growth rates of *csn-1^KO^, csn-4^KO^, csn-5^KO^*, *csn-6^KO^*, and *csn-7^KO^* were slower (Figure 1D). This indicates that these subunits are important for *N. crassa* growth. Unexpectedly, the *csn-3* mutant exhibited hyphal formation and conidiation that was the same as the wild-type strain (Figure 1C). Furthermore, growth of the *csn-3^KO^* strain was slightly faster than that of the wild-type strain (Figure 1D), suggesting that CSN-3 is not a key regulator of growth and development in *N. crassa*. Taken together, these observations demonstrate that the seven subunits of the CSN complex play different roles in the growth and development of *N. crassa*.

### Different CSN subunits differently regulate conidiation rhythms

**Figure 2:**
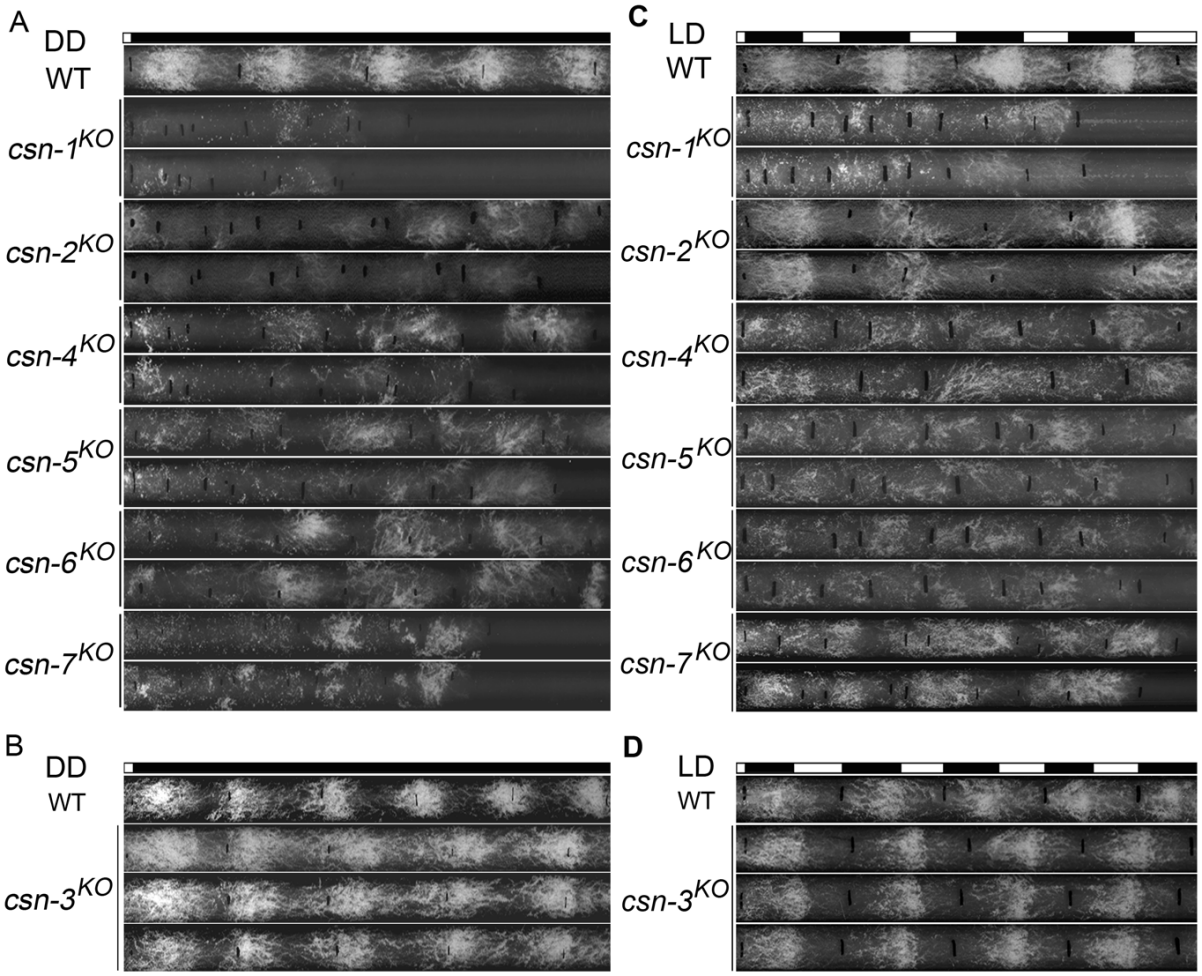
Effect of disrupting different *csn* subunit genes on conidiation rhythms. In DD (A, B) and in LD (C, D). At least four replicates were tested under each condition, and two representatives are shown. The black lines indicate the growth fronts every 24 h.

CSN-2 was found to be a regulator of the *N. crassa* circadian clock [20]. To find out whether other CSN subunits have a similar role, we examined the conidiation rhythm of *csn* knockout mutants by race tube assays. After entrainment by light, the wild-type strain exhibited a robust circadian conidiation rhythm with a period of about 22 hours at 25°C in constant darkness (Figure 2A), while conidiation was very irregular in the *csn-1^KO^*, *csn-2^KO^*, *csn-4^KO^*, *csn-5^KO^*, *csn-6^KO^*, and *csn-7^KO^* strains (Figure 2A), suggesting that these subunits are essential for circadian rhythms. In contrast, the *csn-3* mutant grew faster on race tubes than the wild-type strain, and exhibited a robust and precise period of conidiation identical to that of the wild-type strain (Figure 2B), indicating that the CSN-3 subunit was not essential for normal conidiation rhythms in *N. crassa*. Taken together, these observations demonstrate that, of the seven CSN subunits, all but CSN-3 play an important role in circadian rhythm.

The CSN complex regulates photomorphogenesis in plants [1], and CSN-2 is required for light entrainment of conidiation rhythms in *N. crassa* [20]. To test whether the rest of the CSN subunits have a similar function, we examined the conidiation rhythms of each *csn* mutant in light–darkness cycles (12 h light/12 h darkness). As shown in Figure 2C, the conidiation rhythms of the *csn-1^KO^*, *csn-2^KO^*, *csn-4^KO^*, *csn-5^KO^*, *csn-6^KO^*, and *csn-7^KO^* strains were not entrained by light–darkness cycles, indicating that these six CSN subunits play key roles in light regulation of the circadian clock. As expected, like the wild-type strain, the *csn-3^KO^* mutant could be entrained by light–darkness cycles (Figure 2D), demonstrating that CSN-3 is not required for the light-response process in *N. crassa*. These results further confirm that each CSN subunit contributes unequally to light-dependent processes in *N. crassa*.

**Figure 3:**
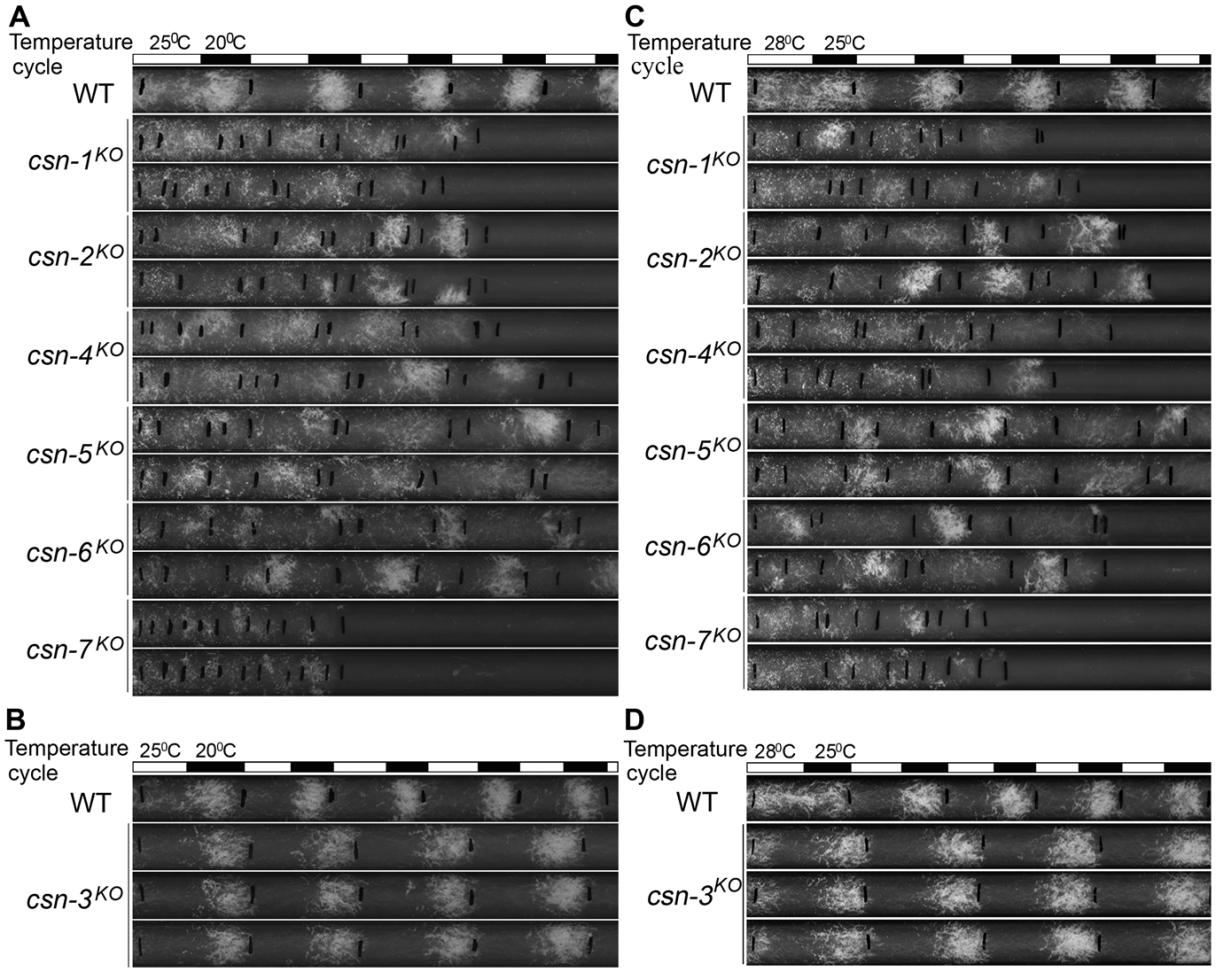
Disruption of different *csn* genes resulted in different responses to temperature cycles in DD. At 25/20°C (A, B) and at 28/25°C (C, D). At least four replicates were carried out under each condition and two representatives are shown. The black lines indicate the growth fronts every 24 h.

Because the temperature-regulated conidiation process is affected in the *csn-2* mutant [20], we examined the responses of the other CSN subunit mutants to temperature entrainment using race tube assays. In 12 h 25°C/12 h 20°C temperature cycles, the conidiation rhythm of the wild-type strain was synchronized, with conidial peaks in the cold phase (Figure 3A). As expected, temperature cycles failed to entrain the conidiation rhythm in the *csn-1^KO^*, *csn-4^KO^*, *csn-5^KO^*, *csn-6^KO^*, and *csn-7^KO^* strains, as was the case with the *csn-2* mutant (Figure 3A) [20], suggesting that these CSN subunits play important roles in the temperature response of *N. crassa*. In contrast, the conidiation rhythm of the *csn-3^KO^* mutant (Figure 3B) could be synchronized by temperature cycles, similar to the wild-type strain, indicating that the CSN-3 subunit is not required for the temperature-response process. Very similar results were seen with 12 h 28°C/12 h 25°C temperature cycles (Figure 3C and 3D). These results suggest that the CSN complex is involved in the regulation of temperature response, and that all of the subunits except for CSN-3 may play key roles.

### Different *csn* mutants differently regulate the neddylation/deneddylation state of cullin proteins

CSN negatively regulates the activities of cullin-based E3 ubiquitin ligases by cleaving the Nedd8 modification from cullins [4], [13]. The distinct phenotypes of each *csn* mutant suggest that they may contribute differently to this particular CSN function. To test this possibility, we examined the neddylation status of three *N. crassa* cullin proteins: Cul1, Cul3, and Cul4. The neddylation status of these cullin proteins reflects the functional activity of the CSN complex in each strain.

**Figure 4:**
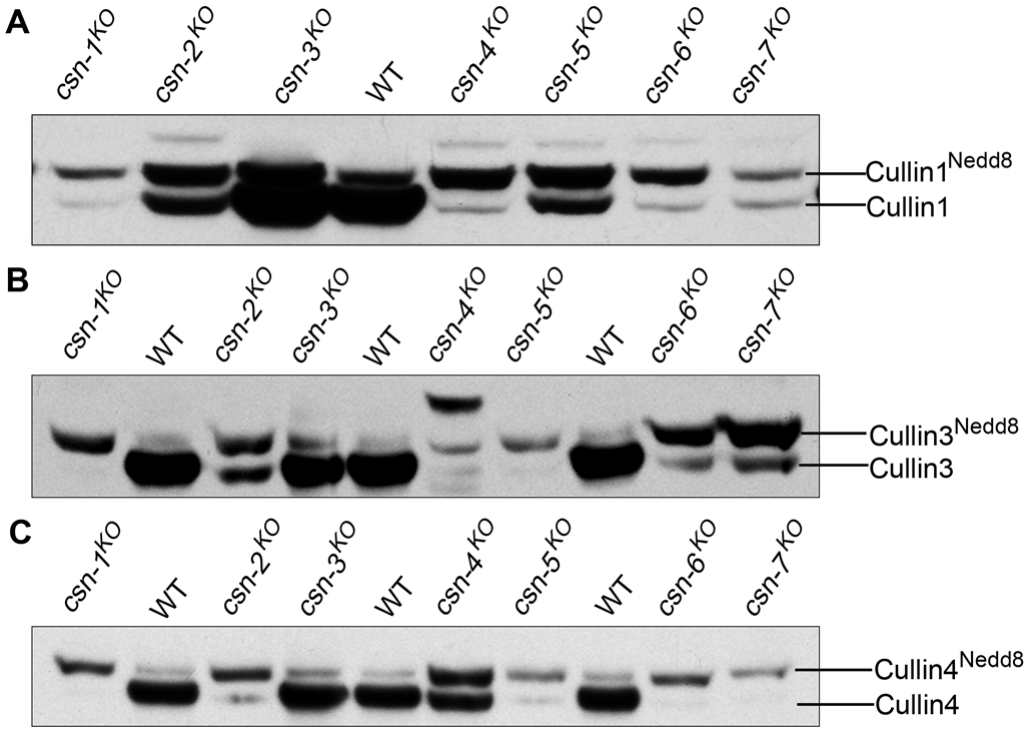
Different cullin neddylation states associated with loss of individual *csn* genes. Western blot analyses with c-Myc antibody of expression profiles of Myc-Cul1 (A), Myc-Cul3 (B), and Myc-Cul4 (C) in the wild-type strain and *csn* mutants.

To monitor the neddylation state of cullins *in vivo*, we introduced a construct expressing Myc-tagged Cul1, Cul3, or Cul4 into the wild-type strain and into each *csn* knockout strain, respectively. Expression of Myc-Cul1, Myc-Cul3, or Myc-Cul4 proteins with the predicted molecular weight was confirmed by western blot analysis. Neddylated Myc-tagged cullins were distinguished from unneddylated Myc-tagged cullins based on their slower migration in an SDS-PAGE gel compared to the unneddylated forms. In the wild-type strain, neddylated Myc-Cul1 (upper bands in Figure 4) represented less than 10% of the total of Myc-Cul1 (Figure 4A). We demonstrated previously that disruption of the *csn-2* gene causes the hyperneddylation of Cul1 [20] (Figure 4A), confirming that normal functioning of the CSN complex is severely impaired in *csn-2* mutants. Similarly, *csn-1^KO^*, *csn-4^KO^*, *csn-5^KO^*, *csn-6^KO^*, and *csn-7^KO^* mutants displayed increased Cul1 neddylation levels (Figure 4A). The Csn5 subunit in *S. pombe* and *D. melanogaster* underlies the Nedd8 isopeptidase activity of the CSN [15]. Our results suggest that the *N. crassa* CSN-5 subunit performed a similar Nedd8 isopeptidase function, and that the other five subunits were also functional subunits for Cul1 deneddylation in *N. crassa*. However, the neddylated/deneddylated Cul1 levels in the *csn-3^KO^* mutant were similar to the pattern in the wild-type strain (Figure 4A), suggesting that CSN-3 is not required for Cul1 deneddylation activity in *N. crassa*. The same Cul1 neddylated/deneddylated patterns were also observed in a *csn-3* deletion mutant (non-band background) derived from FGSC11275 (Fungal Genetics Stock Center) using the Cul1 detection approach described above (unpublished), further confirming that *N. crassa* CSN-3 is not required for the Cul1 deneddylation activity of the CSN complex.

We further examined the neddylation states of two other *N. crassa* cullin proteins, Cul3 and Cul4, in the wild-type strain and *csn* mutants. As shown in Figure 4B, the *csn-3^KO^* mutant had a Myc-Cul3 neddylated/deneddylated pattern that was similar to that in the wild-type strain, whereas in the other *csn* knockout mutants, Myc-Cul3 was hyperneddylated. The neddylated/deneddylated pattern of Myc-Cul4 was similar to the patterns of Cul1 and Cul3 in each strain (Figure 4C). Taken together, these results indicate that CSN-3 is not critical for CSN deneddylation activity, suggesting that CSN-3 is not a key subunit in the CSN complex in *N. crassa*. This molecular evidence, together with the genetic data, strongly suggests that CSN-1, CSN-2, CSN-4, CSN-5, CSN-6, and CSN-7 form the functional core of the CSN complex for cleavage of Nedd8 from cullins in *N. crassa*.

### Different CSN subunits differently contribute to the stability of the SCF^FWD-1^ complex

**Figure 5:**
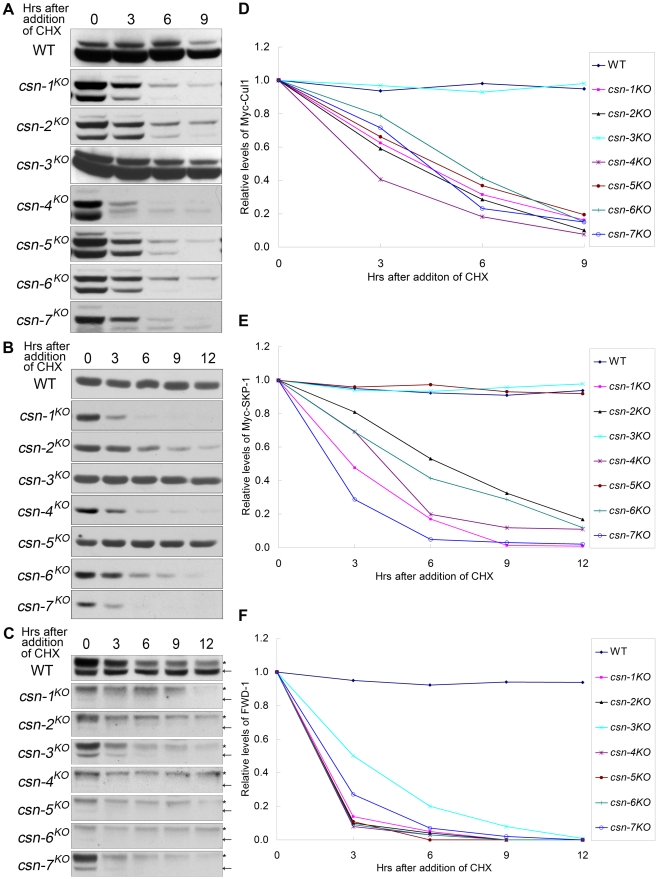
Differing stabilities of components of the SCF complex, Cullin1, and SKP-1 in *csn* mutants. (A, B, C) Western blot analyses showing degradation of Myc-Cul1, Myc-SKP-1, and FWD-1 in *csn* mutants after the addition of CHX (10 µg/ml). Densitometric analyses from four independent experiments showing the degradation of Cullin 1 (D), SKP-1 (E), and FWD-1 (F). Arrows point out FWD-1 protein bands. Asterisks indicate nonspecific bands detected by our FWD-1 antibody.

Recent studies demonstrated that a major function of the CSN complex is to control the stability of E3 ubiquitin ligases *in vivo* [19]–[21]. To determine whether the CSN subunits contribute unequally to the stability control of CRLs in *N. crassa*, we examined the stabilities of Cul1, SKP-1, and FWD-1, the major components of the SCF^FWD-1^ complex, in each of the *csn* strains. As shown in Figure 5A, Myc-Cul1 was very stable in the wild-type strain and the *csn-3^KO^* mutant, with a half-life of more than 9 h in the presence of cycloheximide (CHX). In contrast, Myc-Cul1 in the *csn-1*, *csn-2*, *csn-4*, *csn-5*, *csn-6*, and *csn-7* mutants was unstable, with a half-life of less than 3 h (Figure 5A and 5D). These results demonstrate that the functional core subunits of CSN are responsible not only for Cul1 deneddylation, but also for maintenance of Cul1 stability. In *N. crassa*, SKP-1 is an adaptor protein in the SCF complex that becomes very unstable in *csn-2* mutants [20]. As shown in Figure 5B, Myc-SKP-1 remained very stable in the *csn-3^KO^* mutant and the wild-type strain, with a half-life of approximately 12 h. However, Myc-SKP-1 was unstable in the *csn-1*, *csn-4*, *csn-6*, and *csn-7* mutants, with a half-life of approximately 1.5–3 h (Figure 5B and 5E). Interestingly, as in the wild-type strain and *csn-3^KO^* mutant, the stability of Myc-SKP-1 was not affected in the *csn-5^KO^* mutant (Figure 5B and 5E), indicating that the key deneddylation isopeptidase subunit of the CSN functional core was dispensable for maintaining SKP-1 stability in the SCF complex.

The F-box domain-containing protein FWD-1 is a component of the SCF^FWD-1^ complex; it specifically recognizes phosphorylated FRQ protein and targets it for ubiquitination, which is a key process for circadian clock control in *N. crassa* [20], [29]. As shown in Figure 5C, FWD-1 levels were drastically reduced in the core *csn* subunit knockout mutants and in the *csn-3* mutant, with a half-life of less than 3 h. In contrast, the FWD-1 protein remained very stable in the wild-type strain, with a half-life of more than 12 h (Figure 5C and 5F). Together, these results indicate that the CSN is important for maintaining the stability of F-box domain–containing proteins, such as FWD-1, in *N. crassa*. Although CSN-3 in the CSN complex was not required for deneddylation of Cul1 or for maintaining the stability of Cul1 and SKP-1, it was required for preventing the degradation of FWD-1. These observations suggest that CSN-3 is also required to maintain normal functioning of the intact CSN complex. Taken together, the differing stability of Cul1, SKP-1, and FWD-1 in the *csn* mutants indicates that each subunit of the CSN complex functions differently in maintaining the stability of SCF complexes in *N. crassa*.

### Different contributions of CSN subunits to the stability of Cul3 and associated protein BTB1

**Figure 6:**
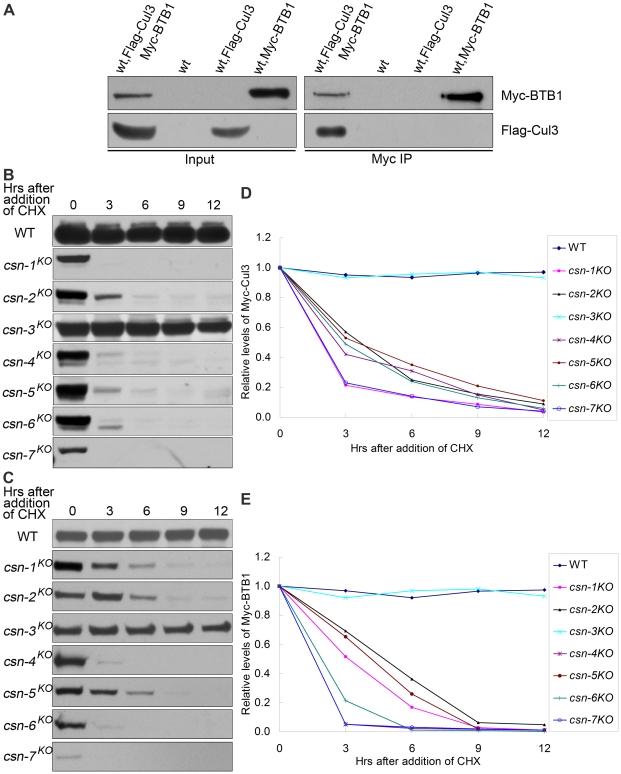
Protein levels of Cul3-based E3 components reduced dramatically in the six core CSN subunit mutants, but not in *csn-3^KO^* mutant. (A) Immunoprecipitation assay confirming the interaction between Flag-Cul3 and Myc-BTB1. Wild-type strain and wild-type strain expressing Flag-Cul3 were used as negative controls. Lysates were subjected to immunoprecipitation with c-Myc antibody, followed by western blot analyses with c-Myc and Flag antibodies. Western blot analyses showing degradation of Myc-Cul3 (B) and Myc-BTB1 (C) in *csn* mutants after the addition of CHX. Densitometric analyses from four independent experiments showing degradation of Cul3 (D) and BTB1 (E).

In *N. crassa*, Cul3-binding proteins have not been reported previously; therefore, we searched for BTB domain protein coding genes in the *N. crassa* genome and found eight predicted proteins with highly conserved BTB domains. To test the interactions between Cul3 and the BTB domain proteins, we created Myc-tagged BTB domain proteins and co-expressed each of them in the wild-type strain with Flag-tagged Cul3. As shown in Figure 6A, BTB1 protein (NCU04838) strongly interacted with Cul3 in the immunoprecipitation reaction, indicating that they may form a Cul3-BTB ubiquitin ligase complex in *N. crassa*.

We next examined whether the stability of Myc-Cul3 was affected in each *csn* mutant. As shown in Figure 6B, Myc-Cul3 was very stable in the wild-type strain and the *csn-3^KO^* mutant, with a half-life of more than 12 h in the presence of CHX. In contrast, this protein was very unstable in the other *csn* mutants, with a half-life of approximately 1.5–3 h (Figure 6B and 6D). These results indicate that the CSN functional core subunits were necessary for maintaining the stability of Cul3 in *N. crassa*.

We then investigated the stability of BTB1 protein in the wild-type stain and *csn* mutants. Myc-BTB1 was very stable in the wild-type strain and the *csn-3^KO^* mutant, with a half-life of more than 12 h after CHX treatment; however, it was very unstable in other *csn* mutants, with a half-life of about 3 h (Figure 6C and 6E). Taken together, these results further demonstrate that the functional core subunits of CSN were important for Cul3 deneddylation and maintaining the stability of the entire Cul3-BTB E3 complex in *N. crassa*.

### Cul4 and DDB1 proteins unstable in all *csn* mutants

**Figure 7:**
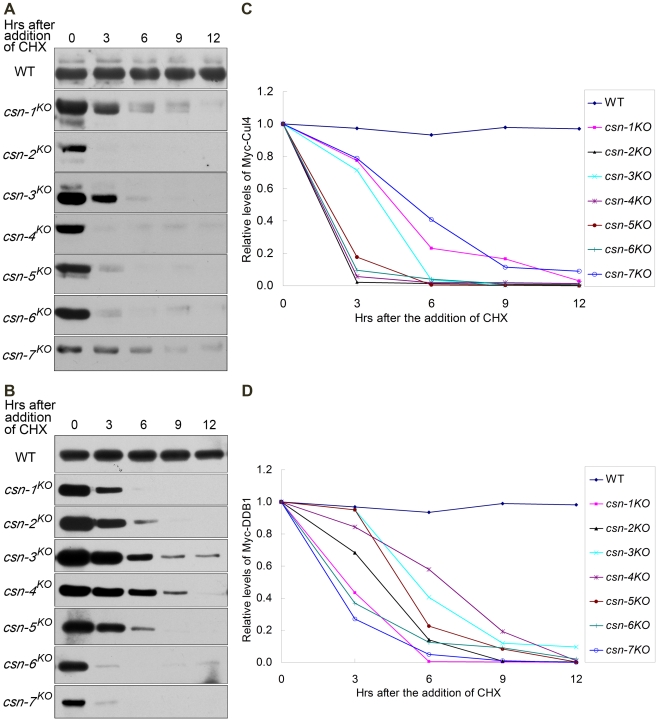
Components of Cul4-DDB1 E3 ubiquitin ligase unstable in all *csn* mutants. Western blot analyses showing degradation of Myc-Cul4 (A) and Myc-DDB1 (B) in *csn* mutants after the addition of CHX. Densitometric analyses from four independent experiments showing degradation of Cul4 (C) and DDB1 (D).

*N. crassa* Cul4 was previously shown to interact with DDB1 [30]. Therefore, we tested the effect of loss of different CSN subunits on the regulation of Cul4-DDB1 E3s. We showed above that the neddylation/deneddylation pattern of Cul4 in the *csn-3* knockout strain was similar to that of the wild-type strain, in which the half-life of Myc-Cul4 was 12 h in the presence of CHX (Figure 7A and 7C). Unexpectedly, Myc-Cul4 was very unstable in all of the *csn* mutants, with a half-life of approximately 3 h after CHX treatment (Figure 7A and 7C). These results suggest that although CSN-3 is not in the deneddylation core of the CSN complex, it was necessary for maintaining the stability of Cul4 in *N. crassa*. We next measured the stability of DDB1 in the wild-type strain and *csn* mutants. As expected, Myc-DDB1 was very unstable in the *csn* mutants, with a half-life of approximately 3 h after CHX treatment (Figure 7B and 7D), while it remained very stable in the wild-type strain, with a half-life of more than 12 h (Figure 7B and 7D). Therefore, unlike in Cul1- or Cul3-based E3 complexes, all of the CSN subunits are required for maintaining the stability of Cul4-DDB1 ubiquitin ligases.

### DDB1- and Cul4-associated factor DCAF11 unstable in all *csn* mutants

**Figure 8:**
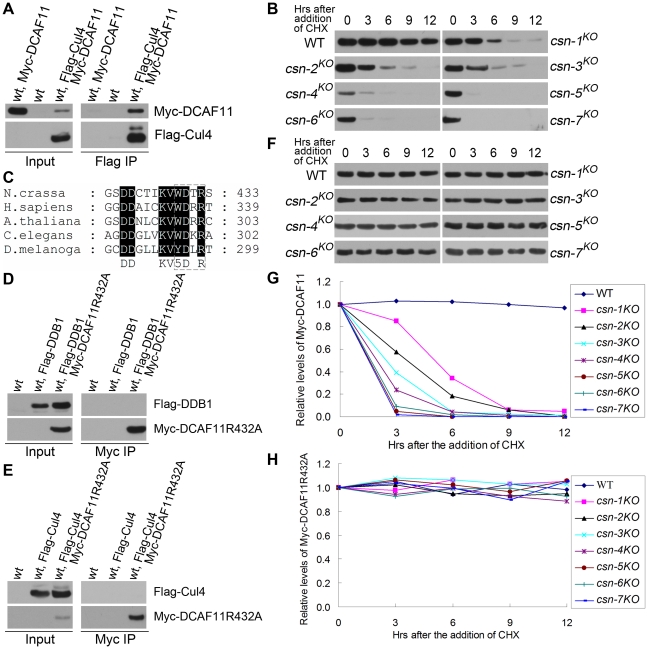
DCAF11 levels drastically reduced in all *csn* mutants, possibly because of rapid autoubiquitination-mediated degradation. (A) Immunoprecipitation assay confirming the interaction between Flag-Cul4 and Myc-DCAF11. Wild-type strain and wild-type strain expressing Myc-DCAF11 were used as negative controls. (B) Western blot analyses showing the degradation of Myc-DCAF11 in *csn* mutants after the addition of CHX. (C) Amino acid alignment of conserved WDXR motifs of DCAF11 homologs. (D) Immunoprecipitation assay with c-Myc antibody showing that Myc-DCAF11R432A failed to interact with Flag-DDB1. (E) Myc immunoprecipitation assay showing that Myc-DCAF11R432A failed to interact with Flag-Cul4. (F) Degradation of Myc-DCAF11R432A expressed in the wild-type strain and all *csn* mutants after the addition of CHX. Densitometric analyses from four independent experiments showing degradation of DCAF11 (G) and DCAF11R432A (H).

We previously showed that in *N. crassa,* DCAF11 is an adaptor protein in a Cul4-DDB1 E3 ligase complex by association with DDB1 protein [31]. In the present study, we performed an immunoprecipitation assay to detect interactions between Myc-DCAF11 and Flag-Cul4. As shown in Figure 8A, Myc-DCAF11 interacted with Flag-Cul4, confirming that it forms an E3 complex with Cul4 and DDB1 proteins. To examine whether the adaptor protein DCAF11 is unstable in the *csn* mutants, we compared DCAF11 degradation rates in the wild-type and seven *csn^KO^* strains. As shown in Figure 8B, DCAF11 was stable in the wild-type strain, as a majority of DCAF11 was still present after 12 h of CHX incubation. In contrast, DCAF11 became undetectable after only 3 h of CHX treatment in the core *csn* mutants and in the *csn-3^KO^* strain, indicating that it was very unstable (Figure 8B and 8G). The accelerated DCAF11 degradation rate in the *csn* mutants was likely due to its increased autoubiquitination, which is counteracted by normal CSN activity. If this is indeed the case, mutation of a conserved arginine in the WDXR motif of DCAF11 (Figure 8C) should disrupt binding of DCAF11 to the Cul4-DDB1 complex, thus preventing its autoubiquitination and degradation. As shown in Figure 8D, interactions between DDB1 and DCAF11 were disrupted by substitution of the conserved arginine with alanine in the DCAF11 WDXR motif. This point mutation also abolished interactions between DCAF11 and Cul4 (Figure 8E). Indeed, Myc-tagged DCAF11 with an arginine-to-alanine point mutation was very stable and accumulated to reach high steady-state levels in all *csn* mutants, including the *csn-3^KO^* strain (Figure 8F and 8H).

Taken together, these data indicate that CSN is important for maintaining the stability of DCAF11 in *N. crassa*, probably by preventing its autoubiquitination. DCAF11 levels were also low in the *csn-3* mutant, indicating that the functional core complex alone was not sufficient to protect Cul4-DDB1 E3 ligase complexes from autoubiquitination and degradation. Because the adaptor proteins FWD-1 and DCAF11 are very unstable in the *csn-3* strain, maintenance of functional E3 ligase complexes may rely mostly on newly synthesized FWD-1 and DCAF11 proteins. These observations suggest that the CSN-3 subunit is also required to maintain the function of intact CSN complexes in protecting cullin-RING E3 ligase adaptor proteins from autoubiquitination.

### Purification and identification of components of the CSN complex in *csn-3^KO^*

**Figure 9:**
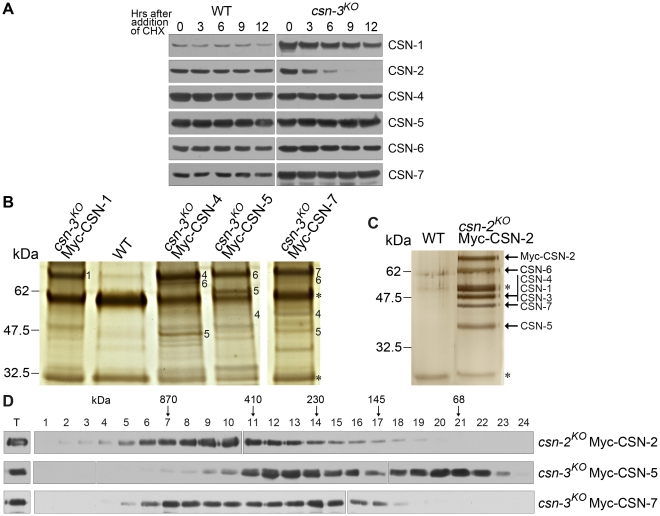
Comparison of CSN assembly in a *csn-3* mutant and a wild-type strain. (A) Western blot analyses showing stability of Myc-His-tagged CSN-1, CSN-2, CSN-4, CSN-5, CSN-6, and CSN-7 in the wild-type strain and *csn-3* mutant after the addition of CHX. (B) Silver-stained SDS-PAGE showing the two-step purification of Myc-His-CSN-1, CSN-4, CSN-5, or CSN-7 in the *csn-3* mutant and the purification of Myc-His-CSN-2 in the *csn-2* mutant (positive control). Wild-type strain was used as the negative control. CSN subunits identified by mass spectrometry analysis are indicated. Asterisks indicate the two IgG bands. 1 for CSN-1 protein, 4 for CSN-4, 5 for CSN-5, 6 for CSN-6 and 7 for CSN-7. (D) The Myc-His-CSN-2 in *csn-2* mutant, Myc-His-CSN-5 or Myc-His-CSN-7 in *csn-3* mutant properly incorporate into CSN complex, but recombinant CSN-7 or CSN-5 form subcomplexes or remained monomer state in the absence of CSN-3.

We next investigated whether loss of the CSN-3 subunit affects the protein levels of other CSN subunits and proper assembly of the CSN complex. Recent studies demonstrated that downregulation of CSN1 and CSN3 causes a proportional reduction in all CSN subunits and a decrease in levels of the holocomplex [32]. We first introduced a Myc-His-tagged CSN-1–, CSN-2–, CSN-4–, CSN-5–, CSN-6–, or CSN-7–expressing construct into a *csn-3* mutant and a wild-type strain, respectively. Western blot analyses showed that Myc-His-CSN-2 became very unstable in the *csn-3^KO^* mutant, with a half-life of less than 3 h in the presence of CHX (Figure 9A). In contrast, Myc-His-CSN-2 was very stable in the wild-type strains, with a half-life of more than 12 h (Figure 9A). Other five Myc-His-CSN proteins remained very stable in the wild-type strain and in the *csn-3^KO^* mutant, with a half-life of more than 12 h (Figure 9A). These results indicate that CSN-3 is required for maintaining the stability of the CSN-2 subunit in *N. crassa*.

Expression of Myc-His-tagged CSN did not affect the phenotype of the *csn-3^KO^* mutant, suggesting that the CSN fusion protein may function similar to the endogenous counterpart subunit. Thus, we used above transformants to purify the CSN complex in the absence of the CSN-3 subunit. Myc-His-tagged CSN-1, CSN-4, CSN-5, or CSN-7 protein was purified on a nickel column followed by immunoprecipitation using a c-Myc monoclonal antibody, respectively. As shown in Figure 9B, several major protein bands were detected in the Myc-His-CSN-4, Myc-His-CSN-5 or Myc-His-CSN-7 sample, but not in Myc-His-CSN-1 sample and the wild-type strain (a negative control). Liquid chromatography–mass spectrometry/mass spectrometry (LC-MS/MS) analysis of excised gel bands led to the identification of CSN-5 and CSN-6 in the Myc-His-CSN-4 purified products, CSN-4 and CSN-6 in the Myc-His-CSN-5 purified products, while CSN-4, CSN-5, and CSN-6 in the Myc-His-CSN-7 purified products, but no CSN-1 and CSN-2 were detected in any of these purifications (Figure 9B). However, there was substantially more CSN-4, CSN-5 or CSN-7 than other CSN subunits in the purification products from *csn-3^KO^*, as revealed by a silver-stained SDS-PAGE gel (Figure 9B); in contrast, the amount of Myc-His-CSN-2 was similar to that of other CSN subunits in a *csn-2^KO^*, QA-Myc-His-CSN-2 transformant (Figure 9C). These results suggest that under this purification condition, CSN-4, CSN-5 and CSN-6 formed a more stable subcomplex, while Myc-His-CSN-7 incorporated subcomplex with CSN-4, CSN-5, and CSN-6. To test whether Myc-His-tagged CSN subunit incorporates into larger molecular mass complex in the absence of the CSN-3 subunit, we performed gel filtration using above purified Myc-His-tagged CSN proteins. As shown in Figure 9D, CSN-7 and CSN-5 fusion proteins were eluted in larger molecular mass fractions and lower molecular mass fractions, while CSN-2 fusion protein remain in high molecular mass fractions. These results confirmed that both Myc-His-CSN-7 and Myc-His-CSN-5 participate in the formation of CSN and the lower molecular mass form. These data indicate that although proper assembly of the *N. crassa* CSN complex is not affected in the *csn-3^KO^* mutant, the amount of CSN complex is decreased and subcomplexes such as CSN4/5/6 and CSN4/5/6/7 are formed in the absence of the CSN-3 subunit, further suggesting that the phenotypes we observed in *csn-3* mutants are due to functional CSN subcomplexes.

## Discussion

CSN is an evolutionarily conserved multifunctional complex found in eukaryotes. In the present study, we used mutant analysis to confirm that the *N. crassa* CSN complex is composed of seven CSN subunits and to demonstrate that each CSN subunit makes a different contribution to CSN function. To the best of our knowledge, this is the first systematic functional analysis of all of the subunits of a complete CSN complex in filamentous fungi. Our results suggest that, of the seven CSN subunits in *N. crassa*, six act as a functional core in cullin deneddylation. Although CSN-3 is dispensable for cullin deneddylation, it plays an important role in maintaining the stability of FBPs such as FWD-1 in SCF complexes, as well as the stability of Cullin4-based ubiquitin ligases and the CSN-2 subunit. In addition, although the absence of CSN-3 did not affect proper assembly of the CSN complex, the amount of functional core CSN complex was reduced and subcomplexes such as CSN4/5/6 and CSN4/5/6/7 were formed.

### Functional core subunits of CSN regulate deneddylation of cullins

In general, CSN complexes contain 7–8 subunits. Accurate assessment of the contribution of individual CSN subunits to the function of the complex as a whole in plants and animals has been difficult, because deletion of any CSN subunit can lead to lethality. Phenotypic features among plants with mutations in different CSN subunit genes are indistinguishable, and plant CSN mutants display almost overlapping misregulation of hormone and other response pathways [33]–[37], indicating that plant CSN subunits function coordinately to support critical deneddylation activity. Fortunately, CSN subunit genes are not essential genes in fungi, such as yeast and *N. crassa*, which are therefore excellent model systems in which to investigate the function of individual CSN subunits.

We successfully created knockout mutants of all of the individual *csn* subunits in *N.crassa*. Of the seven CSN subunits, deletion of CSN-1, CSN-2, CSN-4, CSN-5, CSN-6, or CSN-7 caused similar defects in growth, development, circadian clock, light response, and temperature-entrained conidiation, while deletion of CSN-3 did not cause the defects detected in the other CSN mutants. Similar results were found for other organisms in previous studies. For example, in *S. pombe,* mutations of different CSN subunits cause distinct phenotypes [14]. In *D*. *melanogaster, csn4-* and *csn5*-null flies display different phenotypes [25] and different gene expression patterns [26]. These results, together with the findings of the present study, indicate that different subunits may have different roles in the CSN complex. Consistent with phenotypic analysis results, our molecular analyses demonstrated that CSN-3 was not required for cullin deneddylation, while deletion of any of the other six *csn* genes caused hyperneddylation of all three cullins, indicating that in *N. crassa*, these six CSN subunits were essential for cleavage of Nedd8 from the cullins of CRLs *in vivo*. Nedd8 modification of cullin positively regulates the activities of CRLs. Recent genetic and biochemical analyses demonstrated that the CSN complex is required for removal of Nedd8 from cullin proteins [4], [13], [17], [18]. Thus, the differences in the growth and developmental phenotypes between *csn-3* and the other *csn* mutants are due to the unequal contributions of different subunits to the CSN deneddylation function. Six of the conserved CSN subunits, CSN-1, CSN-2, CSN-4, CSN-5, CSN-6 and CSN-7, likely act as a functional core for CSN deneddylation activity. This idea is supported by the finding that *A*. thaliana plants with an N-terminal deletion in the CSN1 subunit (CSN^CSN1–C231^) exhibit a wild-type pattern of Cul1 neddylation, suggesting that these mutants have normal deneddylation activity [38]. Moreover, the core composition of the CSN complex may also explain why some lower eukaryotes, such as *Candida albicans*, *Cyanidioschyzon merolae*, and *Saccharomyces cerevisiae*, have fully functioning CSN complexes that lack individual subunits [2], [39]. Because CSN is a highly conserved complex that is necessary for regulating the function of CRLs in higher eukaryotes, we propose that a basic functional CSN complex core may exist in these organisms, similar to that found in lower eukaryotes.

### CSN-3 required for maintaining the stability of FBPs and Cul4-DDB1 E3 ubiquitin ligases

CSN3 was first identified in *A. thaliana* as an essential regulator of light-mediated development [1], [40]. Although it is the least-conserved subunit, CSN3 is a component of CSN complexes from plants to mammals. In *A. nidulans*, deletion of genes encoding CSN subunits 1, 2, 4, or 5 resulted in identical blocks in fruit body formation [28]. However, the two nonconserved subunits C (CSN-3) and H (CSN-8) did not interact in a yeast two-hybrid experiment, suggesting that they may require other subunits or posttranslational modifications for stable interactions [28]. We found that deletion of *csn-3* from the genome of *N. crassa* did not affect the neddylation of cullins and slightly increased the growth rate in the mutant strain compared to the wild-type strain. These findings suggest that the role of CSN-3 in the CSN complex is different from those of the other subunits. Hemizygous deletion of human chromosome 17, band p11.2 results in a multiple congenital anomalies/mental retardation syndrome called Smith–Magenis syndrome (SMS) [41]–[43]. The deleted region spans 1.5–2.0 Mb of DNA, which contains about 20 genes, including *CSN3*. To investigate the role of CSN3 in mammalian development and in Smit–Magenis syndrome, *csn3* was disrupted in mice [44]. Interestingly, there are no visible defects in heterozygous *csn3-*disrupted mice, although the protein level was reduced. Embryonic development of homozygous Csn3^−*/*−^ mice is arrested at an early developmental stage [44]. Although Csn2^+*/*−^ heterozygous mice are phenotypically healthy, loss of Csn2 causes embryonic lethality [45]. These studies imply that CSN3, the least-conserved subunit in the CSN complex, may not be important to the function of the CSN complex compared to CSN2 in mice.

In the present study, we demonstrate that the six core subunits of CSN, but not CSN-3, were essential for maintaining the stability of Cul1 in SCF complexes and of Cul3 and BTB proteins in Cul3-BTB E3 ubiquitin ligasess, while five subunits, but not CSN-3 or CSN-5, were required for maintaining the stability of SKP-1 in the SCF complex. This molecular evidence further supports the idea that individual subunits of the CSN complex contribute differently to CSN functions. Consistent with the phenotype of the *csn-3* mutant, CSN-3 does not appear to play an important role in maintaining the stability of these E3s in *N. crassa*. In mutants lacking different CSN subunits, autoubiquitination of FBPs is enhanced, resulting in increased instability. Several FBPs in fission yeast, *N. crassa*, and humans are under the protection of the CSN complex [19], [20], [46]. We further show that FWD-1 stability and levels were drastically reduced in the *csn-3* mutant and other *csn* mutants, confirming that CSN-3 was required for preventing autoubiquitination of FBPs after destruction of their substrates. The modular architecture of SCF complex is apparently shared by several other cullin-RING E3 complexes, such as Cul3, that directly interact with a family of substrate receptors through their common BTB domain, which has a Skp1-like structural fold [12]. Our results show that CSN-3 was not required for maintaining the stability of Cul1, SKP-1, Cul3, or BTB protein in the SCF and SCF-like E3s. We further confirmed that CSN-3 was required for maintaining the stability of CSN-2 in the presence of CHX, and for normal levels of the CSN functional core complex. These data provide evidence that the functional core complex efficiently cleaved Nedd8 from cullins and protected scaffold components of the SCF and SCF-like E3s from autoubiquitination; however, it was not sufficient to protect the FBPs from autoubiquitination. Although FBP levels were also low in the *csn-3* strain, levels of Cul1 and SKP-1 were not affected; some functional SCF complexes formed, most likely by incorporating newly synthesized FBPs, and mediated the degradation of their substrates. These data may explain the normal circadian rhythms that were observed in the *csn-3* mutant, but not in other *csn* mutants.

In contrast, all seven subunits of the CSN, including CSN3, were required to maintain the stability of Cul4-DDB1 E3s in the presence of CHX. The difference in stability compared to SCF and SCF-like E3s is most likely due to architectural differences between Cul4-DDB1 E3s and SCF E3s; recent studies show that DDB1 displays a flexible linkage between the major protein binding domain (BPA-BPC) and the cullin binding domain (BPB) [10], while SCF and SCF-like E3s exhibit more rigid architectures [47]. In support of this possibility, we found that autoubiquitination of DCAF11 was enhanced in all seven *csn* mutants. However, disruption of the interactions between DCAF11 and DDB1 completely abrogated the autoubiquitination and degradation of DCAF11 in *csn* mutants. These data suggest that the instability of Cul4-DDB1 E3s in *csn* mutants could also be due to the strong interactions among their components, which are more powerful than those in the SCF and SCF-like E3s. In this regard, the function of CSN-3 is similar to that of the functional core subunits. These results provide new insight into how CSN functions to protect different CRLs. Based on these data, we speculate that the different subunits play different roles in maintaining the stability of different CRLs, and that the biochemical functions of the CSN subunits are distinct from one another. Although CSN-3 is the least-conserved subunit, it is found in all of the CSN complexes studied to date, from fungi to mammals [28], [40], [44]. Thus, we propose that CSN-3 may have a similar role in regulating the function of CRLs in higher eukaryotes.

## Materials and Methods

### Strains and culture conditions

The *N. crassa* strain 87-3 (*bd*, *a*) was used as the wild-type strain in this study. The *bd ku70^RIP^* strain, which was generated previously [48], was used as the host strain for creating the *csn* knockout mutants. The *csn-2^KO^* and *csn-2^KO^*, *his-3* strains used in the present study were also created previously [20]. The newly created *csn* knockout strains were *csn-1^KO^*, *csn-3^KO^*, *csn-4^KO^*, *csn-5^KO^*, *csn-6^KO^*, and *csn-7^KO^* strains; *his-3* strains were also created for each *csn* deletion. The 301-6 (*bd*, *his-3*, *A*) strain and *csn*, *his-3* strains were the host strains for the *his-3* targeting construct transformation. Liquid culture conditions were the same as described previously [49]. For quinic acid–induced protein expression, 0.01 M QA (pH 5.8) was added to liquid medium containing 1× Vogel's medium, 0.1% glucose, and 0.17% arginine [50]. The medium for the race tube assays contained 1× Vogel's medium, 0.1% glucose, 0.17% arginine, 50 ng/ml biotin, and 1.5% agar.

### Generation of *csn* strains in *N. crassa*

To generate *csn* gene knockout strains, the entire open reading frames (ORFs) of *csn* genes were deleted by replacement with the *hph* gene [51]. The gene replacement cassette containing *hph* was introduced into the *bd*, *ku70^RIP^* strain by electroporation. The transformants with *hph* at the *csn* locus were crossed with 301-6 (*bd*, *his-3*, *A*). Ascospores of the crosses were germinated on plates containing hygromycin and histidine. PCR analyses for *hph* and *csn* the ORF region were used to confirm the *csn* knockout strains.

### Plasmids

Full-length ORFs and the 3′-UTR for Cullin3, BTB1, CSN-1, CSN-4, CSN-5, CSN-6, or CSN-7 protein were amplified from genomic DNA by PCR and cloned into the pqa-5Myc-6His and pqa-3Flag plasmids. The previously constructed plasmids pqa-Myc-Cul1, pqa-Myc-His-SKP-1, pqa-Myc-His-CSN-2 [20], pqa-Myc-His-Cul4, pqa-Myc-His-DDB1, pqa-3Flag-Cul4, pqa-3Flag-DDB1 [30] and pqa-Myc-His-DCAF11 were also used for the *his-3* targeting transformation in 301-6 and *csn^KO^*, *his-3* strains.

### Protein analyses

Protein extraction, quantification, western blot analysis, protein degradation assays, and immunoprecipitation assays were performed as described previously [20], [30]. Western blot analyses using a monoclonal c-Myc antibody (9E10, Santa Cruz Biotechnology) or Flag antibody (F3165-5MG, Sigma) were performed to identify the positive transformants. Immunoprecipitates or equal amounts of total protein (40 µg) were loaded into each protein lane. After electrophoresis, proteins were transferred onto PVDF membrane, and western blot analysis was performed using c-Myc antibody, Flag antibody, or FWD-1 antiserum.

### Purification of Myc-His-CSN proteins from *N. crassa*

The *csn-3^KO^* Myc-His-CSN-1, 4, 5, or 7 strain, wild-type strain (negative control), and *csn-2^KO^* Myc-His-CSN-2 strain (positive control) were cultured for approximately 24 h in constant light (LL) in liquid medium containing QA (0.01 M QA, 1× Vogel's medium, 0.1% glucose, and 0.17% arginine). Approximately 15 g of tissue from each strain grown in LL was harvested. The purification procedure was the same as described previously [20]. Fractions containing purified Myc-His-CSN proteins were immunoprecipitated by adding 30 µL of c-Myc monoclonal antibody–coupled agarose beads (9E10AC, Santa Cruz Biotechnology). The precipitates of Myc-His-CSN samples were analyzed by SDS-PAGE (4%–20% and 15% acrylamide, respectively), which was subsequently silver stained following the manufacturer's instructions (ProteoSilver Plus, Sigma). Specific bands were excised and subjected to tryptic digestion and LC-MS/MS.

### Gel filtration chromatography of Myc-His-CSN-5 or CSN-7 in *csn-3* mutant and CSN-2 in *csn-2* mutant

The protocol of gel filtration chromatography was the same as described previously [52]. Briefly, purified proteins (400 µg) were loaded onto a Superdex 200 (GE) gel filtration column that was equilibrated with 25 mL (150 mM NaCl, 20 mM Tris Cl pH 7.4). The proteins were eluted in the same buffer at a flow rate of 0.3 mL/min. Fractions of 0.4 mL were collected starting from the onset of the column void volume (8.0 mL) and finishing at 18 mL (25 fractions). 20 µL of each fraction were prepared in 20 µL of 2× SDS loading buffer, separated by 7.5% SDS-PAGE, then transferred onto PVDF membrane. Western blot analysis was performed using c-Myc antibody (9E10, Santa Cruz Biotechnology).

## Supporting Information

Table S1 The CSN subunits of *Neurospora crassa*.
(0.02 MB PDF)
