# Supplementary material for: Role of Individual Subunits of the Neurospora crassa CSN Complex in Regulation of Deneddylation and Stability of Cullin Proteins
Source: PLoS Genet. 2010 Dec 2;6(12):e1001232. doi: 10.1371/journal.pgen.1001232 (PMC2996332; doi:10.1371/journal.pgen.1001232)
Supplement: Table S1 — The CSN subunits of Neurospora crassa. (0.02 MB PDF) [file pgen.1001232.s001.pdf]

Table S1. The CSN subunits of *Neurospora crassa*

CSN subunits	NCU Number	Domain	e value	Positions of PCI/MPN domain
CSN-1	NCU00157	PCI	3.0e-19	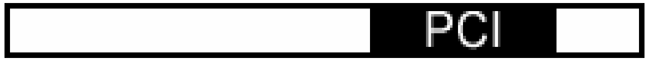
CSN-2	NCU00593	PCI	3.5e-21	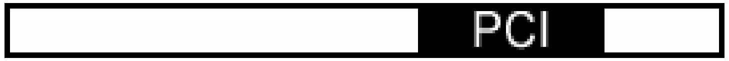
CSN-3	NCU01408	PCI	0.04	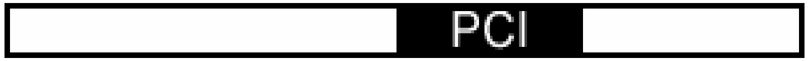
CSN-4	NCU07361	PCI	2.4e-16	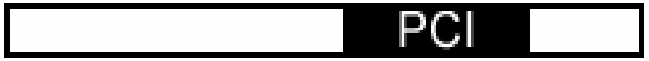
CSN-5	NCU00467	MPN+	1.0e-42	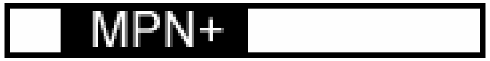
CSN-6	NCU07019	MPN	3.0e-06	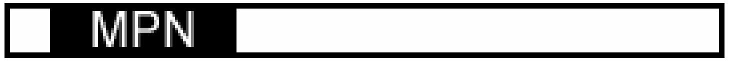
CSN-7	NCU08342	PCI	1.4e-05	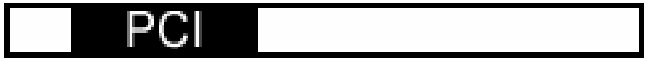

CSN-1-CSN-7 contains PCI/MPN domains (Pfam ID: PF01399/PF01398)
